# A Classroom-Based Intervention for Reducing Sedentary Behavior and Improving Spinal Health: Pragmatic Stepped-Wedge Feasibility Randomized Controlled Trial

**DOI:** 10.2196/65169

**Published:** 2025-02-24

**Authors:** Dominic Fisher, Rentia Maart, Lehana Thabane, Quinette Louw

**Affiliations:** 1 School of Health Professions Faculty of Health University of Plymouth Plymouth United Kingdom; 2 Division of Physiotherapy, Department of Health and Rehabilitation Sciences Faculty of Medicine and Health Sciences Stellenbosch University Cape Town South Africa; 3 Department of Health Research Methods, Evidence, and Impact McMaster University Hamilton, ON Canada

**Keywords:** sedentary behavior, spinal health, classroom-based intervention, sit-stand desks, spinal, African, Africa, primary school, child, youth, randomized controlled trial, RCT, infectious disease, acceptability, data collection, teacher, classroom-based interventions, primary school learners, physical activity, closed-cohort, interview, quantitative data, wearable, wearable sensor, spine

## Abstract

**Background:**

Noncommunicable diseases (NCDs) resulting from sedentary behavior (SB) are adding a further strain on the South African health system, which is already struggling to manage infectious diseases. Some countries have enabled children to reduce SB at school by substituting traditional furniture with sit-stand classroom furniture, allowing learners to interrupt prolonged bouts of sitting with standing without interrupting their school work. Alternating between sitting and standing also benefits spinal health by interrupting prolonged periods of high spinal loading, but no such intervention has been trialed in South Africa. The potential to reduce strain on the health system by reducing the incidence of NCDs and improving spinal health requires further consideration. Before embarking on a large classroom-based trial, it is essential to determine the acceptability of the intervention, its impact on teachers’ practices, and the logistical and pragmatic considerations of data collection.

**Objective:**

This study aimed to assess the feasibility of implementing a classroom-based intervention to reduce SB and improve spinal health in primary school learners, to assess the pragmatics of delivering and adherence to the intervention, and assess the pragmatics of measuring physical activity and postural dynamism data with wearable sensors.

**Methods:**

We used a stratified, closed-cohort, randomized, 2-cluster, stepped-wedge design with a pragmatic approach. One grade 5 and grade 6 class each was recruited from contrasting socioeconomically categorized, state-funded primary schools in the Western Cape province, South Africa. Classroom furniture was substituted with sit-stand desks, and health education and movement videos (HEMVs) were shown during class time. Skin-mounted activPAL physical activity monitors were used to measure SB and postural topography and Noraxon myoMOTION inertial measurement units (IMUs) to measure spinal movement. The study was evaluated for feasibility by tracking school retention, successful delivery of the HEMVs, the use of sit-stand desks, compliance with the wearable sensors, and data accuracy. We deductively analyzed teachers’ interviews and learners’ focus groups using Atlas.ti 9 software. Descriptive analysis of quantitative data was performed using Microsoft Excel.

**Results:**

Cluster 1 withdrew from the study before follow-up SB, postural topography, and spinal movements were measured. All feasibility outcomes, namely (1) classroom retention, (2) delivery of HEMVs, (3) learner and teacher acceptance and usage of sit-stand classroom furniture, (4) 100% compliance with wearing skin-mounted sensors for the duration of the intended measurement period, and (5) minimum 80% eligibility of sensor data gathered included in data analysis, were met in cluster 2. The study found that it is feasible to conduct a larger trial with minor modifications to the methodology.

**Conclusions:**

We recommend a whole-school approach to support the intervention and a monitoring strategy to track the impact of the intervention on the classroom. Furthermore, we recommend contextualized teacher training on how sit-stand desks and HEMVs can be used as classroom management tools.

**Trial Registration:**

Pan African Trials Registry PACTR201811799476016; https://tinyurl.com/y4upoys8

**International Registered Report Identifier (IRRID):**

RR2-10.2196/18522

## Introduction

The prevalence of preventable, long-term noncommunicable diseases (NCDs) presents a serious strain on the South African health care system, already under pressure from the burden of infectious disease management [1]. Although the causes of NCDs are multifactorial, there is consensus that lifestyle, including sedentary behavior (SB), is a major contributor [2]. Any waking behavior with energy expenditure less than or equal to 1.5 metabolic equivalent units while sitting, lying down, or reclining is considered sedentary [1]. Although initial SB interventions were mainly focused on adults, the understanding that SB in adulthood tracks through from childhood and adolescence [3] expanded SB research to include younger populations. Interventions aimed at reducing SB in children have shown promise, with several studies demonstrating good efficacy in reducing SB by educating parents, teachers, and children about the harmful effects of SB [4]. Interventions to address SB initially focused on substituting discretionary screen time with periods of physical activity. However, understanding that the beneficial effects of physical activity, even at the prescribed World Health Organization (WHO) dose, do not undo the detrimental physiological effects of SB [5] has led to the development of interventions to prevent the accumulation of SB.

School-based SB studies have demonstrated high levels of accumulated periods of nondiscretionary sedentary time during class [6]. These high levels of SB are attributable to teachers’ preference that children remain seated during class [7] and environmental barriers to interrupting SB when teachers encourage movement [8]. The conventional classroom environment is not conducive to increasing children’s movement without compromising teaching and learning. As a result, classroom-based SB interventions trialed dynamic sit-stand furniture to overcome the environmental barriers posed by traditional classroom furniture.

Interrupting prolonged sitting bouts by alternating between sitting and standing may have the additional benefit of improving spinal health. Prolonged sitting has been shown to increase axial loading of the spine and increase back muscle activation, which could lead to back pain [9]. Given that spinal pain tracks to adulthood from childhood [7,10] and given the reported high levels of nondiscretionary classroom sedentary time, alternating children’s postural topography may have dual long-term benefits on children’s cardiometabolic and spinal health. It is proposed that alternating between sitting and standing (alternating postural topography) mitigates the effects of prolonged loading in one position by providing periods of relative rest. This approach aligns with the theory of postural dynamism, which encourages regular spinal movement as opposed to maintaining a single preferred spinal position. Altering postural topography may promote spinal health by mitigating axial load on spinal posture and encouraging regular spinal movement. The dual benefit of interrupting prolonged sitting and encouraging transitioning between sitting and standing during class to mitigate the long-term risks to cardiometabolic and spinal health holds significant public health potential in resource-limited contexts, such as South Africa.

Classroom-based interventions comprising dynamic sit-stand furniture, health education, and movement integration that have been conducted in the United States, Europe, Australia, and New Zealand have shown good efficacy in reducing SB without disturbing teaching and learning activities [4]. To the best of our knowledge, no such studies have been conducted in South Africa. Conducting a trial of a classroom-based intervention in South Africa would have to consider a range of contextual factors related to the educational system and socioeconomic inequality, not to mention logistic and pragmatic considerations. Before implementing such a trial, it is important to establish the contextual viability of a previously untested intervention. Given the likely increase in children’s movement in response to a classroom-based intervention and the potential impact on teacher practice, determining the acceptability of the intervention was considered [11,12]. In addition, a pragmatic methodology to measure postural topography, physical activity, and spinal movement needed to be established before being implemented in a large trial. Furthermore, the integrity of data collected in a new environment needed to be assessed. These are all key aspects needed to establish the feasibility of conducting a large trial to assess the beneficial effects of a novel SB intervention in a South African classroom context.

The aim of this study was, thus, to assess the feasibility of implementing a classroom-based intervention to reduce classroom SB and promote spinal health in primary school learners. The study objectives were to assess the pragmatics of delivering and adherence to a classroom-based intervention and assess the pragmatics of measuring physical activity and postural dynamism data with activPAL and Noraxon myoMOTION inertial measurement units (IMUs), respectively. This manuscript focused only on the primary feasibility outcomes. The preliminary findings of the effects will be published subsequently.

## Methods

### Ethical Considerations

This study was granted ethical approval by the Stellenbosch University Health Research Ethics Committee (reference number S17/08/130) and institutional permission by the Western Cape Education Department (reference number 20170525-1279). It was also registered with the Pan African Trials Registry (PACTR201811799476016; International Registered Report Identifier [IRRID] RR1-10.2196/18522).

Prospective participants who expressed interest in participating in the project were provided with study information documents and consent forms. Learners were provided with child assent and parent/guardian consent forms, as well as study information for parents and guardians, which were then collected by the research team upon completion. Before the study began, learners were informed about the intervention and measurement methods. Study data were anonymized by assigning a unique participant identifier to data obtained from participants. As compensation, participants were offered retention of the intervention materials at the conclusion of the project.

### Study Design

A school-based, stratified, closed-cohort, randomized, 2-cluster, stepped-wedge design with a pragmatic approach was used for this study. Participant clusters (school classrooms) were the unit of randomization to determine the order of implementing the intervention. This study report followed the CONSORT (Consolidated Standards of Reporting Trials) extension statement for pilot and feasibility trials (Multimedia Appendix 1) [13]. The protocol for this feasibility trial has been published elsewhere [14]. The CONSORT stepped-wedge flow diagram (Table 1) for cluster randomized trials [15] shows the timing of crossover from control to intervention conditions.

**Table 1:** Stepped-wedge cluster RCT^a^ diagram of the study sequence.

Cluster	Randomized transition from usual classroom conditions to intervention condition					
	Weeks 1-2 (April 23-May 10, 2018)	Week 3 (May 11-16, 2018)	Weeks 4-5 (April 23-May 10, 2018)	Weeks 6-14 (May 14-August 10, 2018)	Weeks 15-16 (August 13-24, 2018)	July 31-August 15, 2019
Cluster 1, Q5^b^ classroom	Usual conditions	Baseline measurement and introduction of intervention	Intervention	Intervention	Withdrawal from study and qualitative interviews	Usual conditions: withdrawal from study
Cluster 2, Q3^c^ classroom	Usual conditions	Usual conditions	Baseline measurement and introduction of intervention	Intervention	Follow-up measurement and qualitative interviews	Follow-up measurement and qualitative interviews

^a^RCT: randomized controlled trial.

^b^Q5: quintile 5.

^c^Q3 quintile 3.

The stepped-wedge design was used to allow the intervention to be evaluated within the bounds of the logistical constraints and context of each cluster [16]. Given the limited availability of measurement equipment, the stepped-wedge design allowed sequential baseline and follow-up measurements. Furthermore, given the evidence of the effectiveness of the mode of intervention in other contexts [4], and other classroom-based interventions, the stepped-wedged design allowed both clusters to receive the potential benefit of the intervention.

### Study Setting

The study was conducted in the Western Cape province of South Africa, a region of broad language, cultural, and socioeconomic diversity. In South Africa, publicly funded (state schools) are categorized into quintiles according to socioeconomic factors, including income, literacy, and employment rates of the surrounding community [17]. Lower-quintile schools are more resource limited than upper-quintile schools and thus receive greater state funding per registered learner. In the metro central district, there are fewer lower-quintile schools, and most schools fall under the quintile 5 (Q5) category.

### Sampling and Recruitment

To ensure a diverse range of contextual factors, primary schools from quintile 3 (Q3) and Q5 were selected exclusively from the central metro district of the Western Cape Education Department. The rationale was that if it were deemed feasible to conduct a future trial in the more challenging, resource-strained context, it would be feasible in less challenging, adequately resourced contexts. A downloaded list of publicly funded schools in the central metro district from the Western Cape Education Department website was delimited to school categories Q3 and Q5. School principals, stratified according to Q3 and Q5 schools, were randomly contacted by the principal investigator via telephone or email for recruitment. Afterward, the principals nominated grade 5 or 6 teachers to attend an information session.

### Sample Size

A sample size calculation was not performed as per the CONSORT extension statement for feasibility studies [18]. The sample target was to include a wide range of contextual factors relevant to the feasibility outcomes. The diversity between the clusters (namely classrooms from Q3 and Q5 schools) was considered sufficient to provide a range of considerations to assess the feasibility of the intervention and the pragmatics of data collection. The individual participants selected for measuring physiological outcomes (SB and postural dynamism) were randomly chosen from the class list provided by the class teacher. An equal number of male and female learners were sampled.

### Intervention

The aim of the intervention is to improve the long-term cardiometabolic and spinal health of the public by reducing the accumulation of prolonged SB that has been shown to be pervasive in schools. Providing learners with an opportunity to transition between sitting and standing during class time helps arrest sedentary physiology and provides respite from prolonged axial spinal loading. The study intervention comprised a novel height-adjustable sit-stand desk and a playlist of 9 health education and movement videos (HEMVs). The study authors (DF and QL) contributed to the development of the intervention. The design concept was informed in part by a systematic review of the efficacy of classroom-based interventions to improve spinal health and reduce the SB of schoolchildren [4], as well as a qualitative study of educators’ perceptions of learners’ movement during class time conducted in the Western Cape province, South Africa [7]. The systematic review provided compelling evidence for the efficacy of classroom-based interventions that include alternative, sit-stand classroom furniture and health education to improve spinal health and SB outcomes.

#### Sit-Stand Desks

The research team considered the sit-stand desks available in South Africa prohibitively expensive and thus unsuitable for implementation in the study. An innovation team developed a novel, multifunctional, height-adjustable, sit-stand classroom desk called the KUZE (Multimedia Appendix 2) for this study. Learners and teachers were shown how to select the correct height for sitting and standing. All the usual classroom chairs were removed and replaced with KUZE sit-stand desks during the study period.

#### Health Education and Movement Videos

The innovation team comprising the authors, professional video content creators, and a teacher who advocates for using body movement in teaching mathematics developed a series of HEMVs for this study. Learners were instructed to follow the videos, which included an interactive component requiring them to solve simple arithmetic problems using corresponding body movements (Multimedia Appendix 3).  The teachers were handed the HEMVs on a mobile external hard drive and, after discussions with the researchers, were given the freedom to develop their own strategies for playing the videos during class time. The videos are available to view on YouTube [14].

#### Control Conditions

The control conditions were the usual classroom conditions. Cluster 1 and cluster 2 classrooms had similar dimensions. The cluster 1 classroom furniture comprised metal framed, wooden, all-in-one, tandem desks that fit 2 learners abreast (Multimedia Appendix 4). The cluster 2 classroom furniture had a combination of single and double metal-framed wooden tables with accompanying plastic-molded chairs (Multimedia Appendix 4). The HEMVs were not played during control conditions.

### Pragmatics of Physiological Data Collection Using Wearable Sensors

#### Physical Activity Monitoring: activPAL

Participants’ classroom physical activity and postural topography (sitting, standing, stepping. and sit-to-stand transitioning) were measured using activPAL3 microsensors (PAL Technologies). In the past decade, activPAL sensors have been widely used to objectively measure physical activity [19-21]. Sensors were applied to participants before the start of lessons and removed after the end of lessons on physiological data collection days. The sensors were attached to the anterior right thigh with a waterproof nitrile sleeve and Opsite dressing, as prescribed by the user manual. Data logged on the sensors were downloaded into a secure file at the end of each day.

#### Spinal Movement: Noraxon myoMOTION Inertial Measurement Units

Postural dynamism was measured using Noraxon myoMOTION IMUs. Wearable IMUs allow for the assessment of postural dynamism in an ecologically valid setting of the classroom. The IMUs combine on-board triaxial gyroscopes, accelerometers, and magnetometers for accurate sensor orientation tracking [22]. IMUs were attached to the head with an elasticated Velcro belt and to the neck, thorax, and sacrum directly to the skin using double-sided tape.

### Description and Measurement of Feasibility Outcomes

The feasibility outcomes include retention of the study, fidelity to the intervention (acceptance, and usage of the KUZE sit-stand desks, delivery of the HEMVs), and integrity of the physiological data collected (compliance with wearing the activPAL and IMU sensors). The success indicators (set a priori) and methods of measuring the 5 feasibility outcomes are outlined in Table 2.

**Table 2:** Summary of feasibility outcomes, success indicators, and measurement methods.

Feasibility outcome	Success indicator	Measurement method
Cluster retention	Both clusters remained in the study until follow-up measurements were obtained.	Retention rate recorded on the project management record Qualitative feedback obtained from teachers regarding retention
Delivery of HEMVs^a^	Teachers developed a routine of playing the HEMVs and adhered to the routine.	Qualitative feedback obtained from teachers and learners at the exit interview
Acceptance and usage of the KUZE desk in the classroom	Learners and teachers accepted and used the KUZE as classroom furniture for the entire study period.	Retention rate recorded on the project management record Qualitative feedback obtained from teachers and learners regarding acceptance of the KUZE
Compliance with wearing activPAL and IMU^b^ sensors	There was 100% compliance with wearing activPAL sensors and IMUs for the duration of the intended measurement period.	Recorded on the project management record
Integrity of physical activity and postural dynamism data	Of all data captured by activPAL and IMU sensors, 80% were eligible for inclusion in the analysis.	Recorded on the project management record

^a^HEMV: health education and movement video.

^b^IMU: inertial measurement unit.

Feasibility outcomes were measured using both qualitative and quantitative methods. For the qualitative measurement of the feasibility outcomes, individual depth interviews (IDIs) with the class teachers and focus group discussions (FGDs) with a subgroup of learner participants from each cluster were conducted at the end of the intervention period. The success criteria of the feasibility outcomes were used as the framework for the IDIs and FGDs. The quantitative methods encompassed the physiological data obtained from the activPAL and IMU sensors, as well as estimates of retention and compliance, which were monitored by the researcher.

#### Interpretation of Feasibility Criteria

The success criteria set a priori were:

- Continue with a large pilot/trial if all 5 success criteria are met in both clusters;
- Make minor modifications to the protocol if 3 or more criteria are met in both clusters before continuing with a pilot/trial; or
- Make significant protocol modifications if 2 or less criteria are met in both clusters [18].

### Data Analysis

#### Qualitative Analysis

A deductive analysis of data from IDIs with teachers and FGDs with learners was conducted to determine the feasibility outcomes and success indicators. The qualitative feedback sessions were recorded and transcribed in full and then analyzed using Atlas.ti 9, a computer-assisted qualitative data analysis software program that facilitates the organization, coding, analysis, and visualization of data [23]. Learner and teacher responses were grouped into corresponding themes related to classroom retention, intervention delivery, acceptance, and usage of the KUZE. At the end of the intervention, 2 FGDs per cluster were conducted. The focus group comprised both individuals who provided physiological data and those who were only exposed to the intervention but did not provide physiological data. Verbatim representative quotes of participant responses were provided.

#### Quantitative Analysis

Categorical data were analyzed descriptively and presented as percentages. The number of participant classes (clusters) that remained in the study for the duration of the study was documented and presented as a percentage of the total participant classes that enrolled in the study. The proportion of data used in the analysis compared to data collected was presented as a percentage. All analyses were performed using Microsoft Excel.

## Results

### Demographic Characteristics

**Figure 1:**
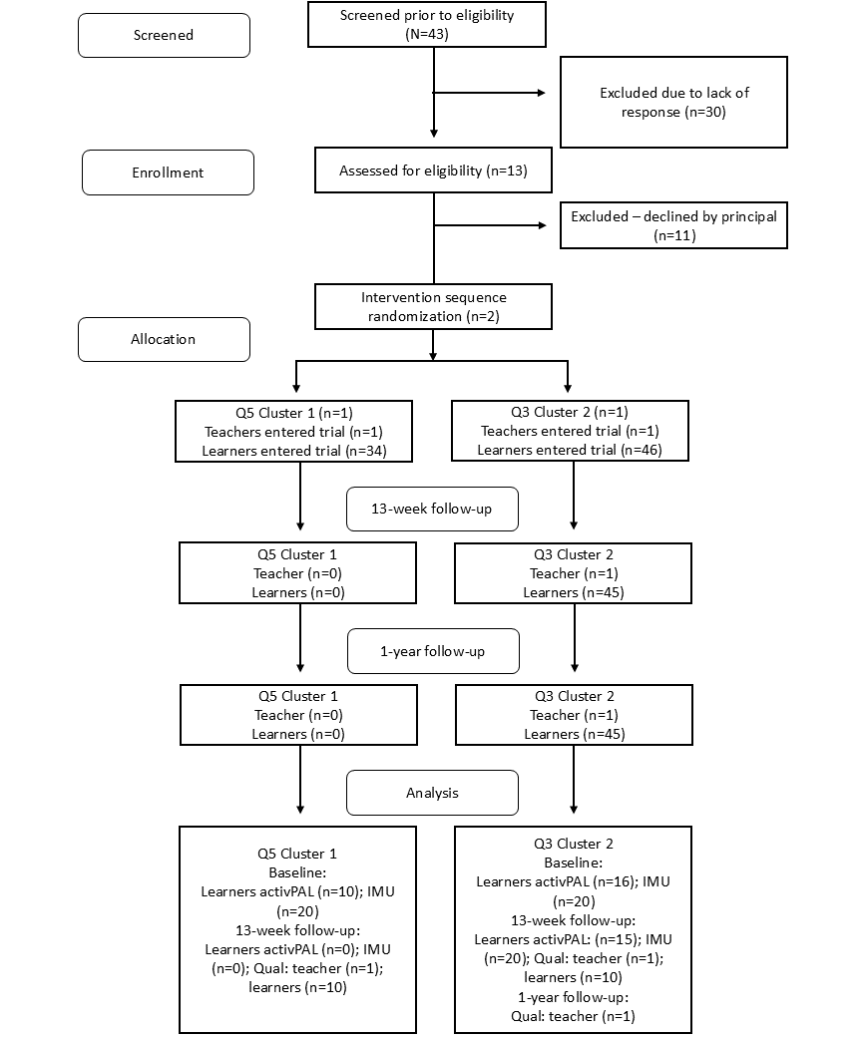
CONSORT flow diagram of the feasibility RCT. CONSORT: Consolidated Standards of Reporting Trials; IMU: inertial measurement unit; Q3: quintile 3; Q5: quintile 5; Qual: qualitative; RCT: randomized controlled trial.

The CONSORT flow diagram of the study is illustrated in Figure 1. The baseline demographic characteristics of participants were similar across clusters. The cluster 1 (Q5) school comprised a grade 6 class, while cluster 2 (Q3) was a grade 5 class. Thus, the ages of the learners in this study were 10-11 years. An equal number of male and female learners made up the study sample.

### Feasibility Outcome 1: Cluster Retention

The cluster 1 (Q5) teacher withdrew her consent to participate in the study after the 13-week intervention and before physiological follow-up measurements were taken. However, the teacher and learners participated in the IDI and FGDs, respectively. Cluster 2 (Q3) remained in the study throughout the study period. Table 3 demonstrates at which stage the cluster 1 teacher withdrew.

**Table 3:** Components of retention for clusters 1 and 2.

Cluster	Recruitment	Baseline measurements	Intervention	Follow-up measurements	Qualitative interviews
1	✓	✓	✓	Withdrew	✓
2	✓	✓	✓	✓	✓

#### Qualitative Findings

Based on the qualitative findings, the reasons for withdrawal from the study were analyzed into 3 themes: the way access to the classroom was negotiated; the teacher’s preference for not being video-recorded during data collection; and the perceived disruption to teaching and learning by the study.

#### Access Negotiation

The same strategy was used to recruit both clusters. Negotiation of access to the classroom was conducted via the school principal and the original teacher (cluster 1). However, a last-minute substitute teacher was employed immediately before the commencement of the study. The school principal discussed the project and its requirements with the substitute teacher. However, the substitute teacher felt that she was not adequately informed of the full extent of the study, despite receiving the study information material.

So, I didn’t really know. So, I was like, okay, I’ll wait for you (the researcher) to come; maybe you’ll tell me, and then suddenly, it was a whole setup in my class, and I was, like, what is going on?

Teacher, cluster 1

The teacher in cluster 2 was satisfied with the recruitment strategy.

Yes. It was the right way. Starting with the principal of course…the principal would never decide on her own. She had to go to the teachers and bring this to the teachers and hear what they say.

Teacher, cluster 2

#### Preference for Not Being Video-Recorded

The cluster 1 teacher was uncomfortable about being recorded on video. She reported that she was self-conscious about being on camera, which influenced her interactions with the learners. The teacher in cluster 2 did not have objections to the use of video for postural dynamism data collection.

Well, I mean, having someone basically watching you (laughing). You don’t really have freedom with your kids because you say something, but you get recorded on video.

Teacher, cluster 1

#### Perception of Disruption to Teaching and Learning

The cluster 1 teacher felt that the intervention (KUZE) interfered with her preferred way of arranging the classroom furniture.

And I found that because the way my class is set out, I put my class when it was rows, so before, I had groups, but then when the desk came, I only could have rows because that was the only thing that could fit in my class.

Teacher, cluster 1

To standardize the orientation of learners for postural dynamism measurement, the learners were required to sit facing the front of the classroom. The teacher reported that the learners often had to be moved from their usual desks, and this impacted interpersonal dynamics with the person next to them.

Maybe that, but also, as you know my class, I had groups. So, you needed your kids to face the front. So, that also caused moving around and disruptions, like this child don’t want to sit next to that child, but yet, they had to because...to accommodate the project.

Teacher, cluster 1

The teacher was concerned that disruptions to teaching and learning would impact the learners’ academic performance and decided it was best to withdraw from the study.

I don’t think my kids can afford to have all that interruptions, especially during the second term, when we have exams, and the third term, when we have systemics. So, I needed to be focused all the time. So, having the disruption was just a lot to manage.

Teacher, cluster 1

### Feasibility Outcome 2: Delivery of HEMVs

Both teachers reported having developed a routine for playing the HEMVs during class. The cluster 1 teacher played the HEMVs intermittently during the day, while the cluster 2 teacher used the videos as a classroom management tool to his advantage. He played the videos at the start of the periods to engage learners and prepare them for the lesson.

So, what I did is the morning I played them one, in the beginning of the day and before first break. Then, second break, I don’t have them, so...they go for 2 hours…they go somewhere else (to a different classroom), and then maybe a video at the end of the day.

Teacher, cluster 1

Because of the start of the period, I want them to bring their minds before doing maths…it’s like an exercise where everybody would feel right for the lesson…We didn’t do that a lot every day. Most of the time, I did the videos when it’s my first period in my class.

Teacher, cluster 2

### Feasibility Outcome 3: Acceptance and Usage of the KUZE Desk in the Classroom

Teachers and learners in both clusters accepted the KUZE as the classroom furniture for the duration of the intervention period. The cluster 1 teacher allowed the learners to stand during groupwork activities for specific subjects and during times where independent work was required.

I think the desk was helpful in that situation, yes, where they had their own work to do without having extra work on the board or looking at the board. So, when they had independent tasks like that, then I think it was okay to be standing, but also not for a long period of time because they tend to move around and they’re free to walk around.

Teacher, cluster 1

I think it was actually amazing for me not to be sitting...just sitting there like a stick insect the whole time, and sometimes, you can’t even concentrate in class because your head is down. But you’re active from those desks you brought us (KUZE).

Learner, cluster 1

Learners in cluster 2 were allowed to use their discretion in whether to sit or stand while completing their work and to break up periods of sitting. At the end of the intervention period, the KUZE desks were removed from cluster 1 and the usual classroom furniture was returned. The cluster 2 teacher requested that the KUZE remain as the standard classroom furniture in his class beyond the completion of the study. When asked about whether they wanted to return to using their usual classroom furniture, learners from cluster 2 chose to keep the KUZE furniture.

I think I was going to use it for the new learners maybe next year. Because as I can see now, the other classes, they see these desks. Now they are also curious...

Teacher, cluster 2

I want you to keep the cosi (KUZE) chairs for us…and our teacher said the cosi (KUZE) chairs are good for us so that we can stand.

Learner, cluster 2

The teacher in cluster 2 reported that the KUZE desks provided a valuable advantage of more space to access all the areas of the classroom.

The advantage they (KUZE desks) give me is that in that classroom there is space; it’s spacious, you see. I can now move in between them…I am able to move around, looking at their books.

Teacher, cluster 2

### Feasibility Outcome 4: Compliance With Wearing activPAL and IMU Sensors

There was 100% compliance with wearing activPAL sensors and IMUs. No learners reported any adverse skin reactions to wearing the activPAL sensors at any stage of the study. During the 13-week follow-up measurement, 10 participants reported mild head discomfort from the elasticated Velcro strap used to secure the head IMU. In all cases, discomfort was resolved by readjusting the strap. Thereafter, the learners were able to return to class and resume learning activities. The mild discomfort that learners reported from the head strap had resolved before they were allowed to return to the class to complete the period of data collection. All learners selected for physiological measurements complied with wearing the activPAL sensors and IMUs throughout the study.

### Feasibility Outcome 5: Integrity of Physical Activity and Postural Dynamism Data

A low rate of data loss was recorded for physical activity and postural dynamism (Table 4). An average of 94.64% of recorded SB and spinal movement data had sound integrity and was considered eligible for inclusion in the analysis for the secondary objectives. The rate of data integrity met the a priori target of 80%. Data loss from wearable sensors was attributable to technical problems during downloading to the project computer or due to batteries running out of charge.

**Table 4:** Data integrity from wearable sensors.

Measurement period	Cluster 1		Cluster 2	
	activPAL (%)	IMU^a^ (%)	activPAL (%)	IMU (%)
Baseline	87.23	92.86	98.48	100.00
13 weeks	Withdrew	Withdrew	98.48	95.00
1-year follow up	Withdrew	Withdrew	83.75	Not measured

^a^IMU: inertial measurement unit.

### Summary of Feasibility Outcomes

Three of the feasibility outcomes were achieved in 3 criteria in both clusters. As cluster 1 was not retained in the study, the acceptance and usage of the KUZE could only be assessed partially through qualitative feedback and was thus categorized as “partially met.” All 5 feasibility outcomes were achieved in cluster 2 (Table 5).

**Table 5:** Achievement of success criteria.

Feasibility outcome	Success indicator	Outcome	
		Cluster 1	Cluster 2
Classroom retention	Both clusters remained in the study until follow-up measurements were obtained.	Unmet	Met
Delivery of HEMVs^a^	The teacher developed a routine of playing the videos and adhered to the routine.	Met	Met
Acceptance and usage of the KUZE desk in the classroom	Learners and teachers accepted and used the KUZE as classroom furniture for the entire study period.	Partially met	Met
Compliance with wearing activPAL and IMU^b^ sensors	There was 100% compliance with wearing activPAL sensors and IMUs for the duration of the intended measurement period.	Met	Met
Integrity of physical activity and postural dynamism data	Of all data captured by activPAL and IMU sensors, 80% were eligible for inclusion in the analysis.	Met	Met

^a^HEMV: health education and movement video.

^b^IMU: inertial measurement unit.

## Discussion

### Principal Findings

This study effectively assessed the feasibility of implementing a classroom-based intervention to reduce classroom SB and promote spinal health in primary school learners in the Western Cape province of South Africa. Our findings showed that the a priori success criteria to decide to continue with a large trial were met. Having met 3 feasibility outcome success criteria (delivery of HEMVs, compliance with wearing physical activity and postural dynamism sensors, and integrity of the data gathered) in both clusters indicates that with minor modifications to the study methodology, it is feasible to progress to a larger trial. Despite this positive finding, reduced cluster retention may be a risk of a larger trial. Therefore, strategies to enhance retention are an important consideration for a larger trial.

Individual teacher-related aspects should be considered in retention strategies. The last-minute substitution of the teacher meant that the research team was unable to establish a satisfactory level of engagement, rapport, and support before the study started. This may have led to a negative attitude toward the study, which was compounded by her perceived loss of autonomy due to the study-related change to the classroom furniture and configuration. Holistic support of teachers is important in classroom-based movement integration interventions [24] to maintain adequate levels of engagement and support throughout the process of the study. Such a holistic whole-school approach that includes senior school management and other school stakeholders has gathered traction in interventions to increase physical activity in UK primary schools [25]. However, such a whole-school approach will need to be contextualized for the South African school system, considering the scarce track record of implementation of similar interventions. Given the importance of teachers in implementing the study and the dynamic nature of school settings, the design of a future trial should incorporate pragmatism to ensure that timely and appropriate adaptation is possible throughout the study [26].

Although not retaining cluster 1 (Q5) was disappointing, it provides a valuable learning opportunity to inform a larger trial. It is, therefore, recommended that a customized school context–specific program of engagement, awareness, and involvement in the study precede a future trial to improve participants’ experience of participation [27] and to optimize its acceptability and efficacy [28]. Prior teacher involvement and program monitoring may also provide an opportunity to codevelop cluster retention strategies for implementation in a future trial; aid the development, implementation, and adaptation of the intervention; and track cluster progress against study goals [29]. Cocreation of school-based health interventions has been shown to improve the feasibility of interventions in the complex environment of the school context [30]. Including teachers in the development and implementation of an intervention can leverage their expertise of the classroom environment and enable them to find solutions to concerns about the potential classroom disruptions and reconfiguration of classroom furniture. Raising awareness about the study with a view to including teachers to codevelop the intervention may be important considerations for a future trial, given the sizeable investment requirement in this emerging area of research in South Africa.

Despite no previous classroom-based interventions including sit-stand desks in South Africa, the KUZE desk was well accepted by teachers and learners. Teachers recognized the positive impact of allowing learners to stand on their ability to work independently and on their concentration after prolonged periods of sitting. However, some concerns were raised about disruption to teaching and learning and loss of control of the classroom, which presents a potential threat to the rollout of a future trial. Similar concerns were voiced by teachers in a previous UK-based trial [11]. These concerns are reasonable, given the longstanding tradition and practice of enforcing learners to remain seated during class time. Interrupting periods of sitting with standing tasks challenges teachers’ practice and propensity to enforce sitting during teaching and learning activities. In a study of teachers’ perspectives of learners’ movement during class, participants reported that they believed that sitting enhances learners’ concentration [7] and were unaware of the evidence that interrupting prolonged sitting may enhance their concentration and engagement with cognitive tasks [31,32]. Teachers in the study by Fisher and Louw [7] associated enforcing that learners remain seated with teachers’ maintaining control of the classroom and their level of classroom management skill and teaching pedagogy. It may be prudent to emphasize the benefits of interrupting prolonged sitting for learners in future teacher training and support as part of a future trial of the intervention.

The activPAL sensor wear protocol used in this study did not appear to impact the logistics or integrity of the physical activity data collected. The decision to apply and remove the activPAL sensors daily was mainly motivated by not wanting to risk losing the expensive devises. The research team also considered the ethics of the potential safety risks study participants would bear by wearing expensive devices in their communities without any mitigating measures in place. Most studies of free-living physical activity using activPAL adopt a 24-hour, 7-day wear protocol [33]. However, this wear protocol has drawn some criticism, with evidence of dose–response noncompliance with wear protocols approaching the 7-day period [34]. Reports of skin irritation caused by perspiration, particularly in warm, humid conditions, have threatened study participants’ compliance with wearing the sensors [35]. The fact that no skin irritation was reported by study participants justifies the rationale for the wear protocol. If this activPAL wear protocol was adopted in a future study, it may be useful to determine the validity of the single wear protocol compared to the 24-hour, 7-day wear protocol.

### Study Limitations

The study was limited to schools in a specific geographical and socioeconomic context. Therefore, the findings may not be broadly generalizable. The interpretation of some findings was limited by the suboptimal retention of 1 cluster. The study was also limited to specific preset feasibility criteria and important implementation factors; for example, teacher and school dynamics were not considered. The feasibility study’s data are also arguably too limited for sample size considerations for a larger trial, and further research is required.

### Conclusion

This study addressed the uncertainty of the acceptability of a classroom-based SB and spinal health intervention, its impact on teachers’ practice, and the logistical and pragmatic considerations of data collection in a previously unresearched setting. Having established the feasibility of some aspects of the intervention in the local context, the acceptance of sit-stand desks remains questionable and presents a potential risk to the success of a larger trial. Although teachers and learners from both clusters accepted and used the intervention classroom furniture for the duration of the intervention period, the feedback from the cluster 1 teacher raises some uncertainty. However, these concerns can be addressed, as described in the recommendations. The results of this study indicate that minor revisions to the current methodology are required to conduct a large cluster randomized controlled trial (RCT) of an intervention aimed at reducing SB and improving spinal health in primary schools in the Western Cape province.

Recommended changes to the methodology include:

- A holistic whole-school approach to the intervention, including all grade teachers and school management, to support implementation of the intervention, particularly playing HEMVs
- A monitoring strategy to track the impact of the intervention on the classroom, teacher, and school’s environment, as well as the ability to adapt and implement pragmatic strategies to enhance retention
- Inclusion of teachers in the cocreation, monitoring, and implementation of the intervention to improve the feasibility of a future trial
- Contextualized teacher training on SB and how sit-stand desks and HEMVs can be used as a classroom management tool

## Appendix

Multimedia Appendix 1 CONSORT (Consolidated Standards of Reporting Trials) extension statement for feasibility of sedentary behavior (SB) and spinal health.

Multimedia Appendix 2 Learner using KUZE for sitting and standing.

Multimedia Appendix 3 Still from a health education and movement video (HEMV).

Multimedia Appendix 4 Cluster classroom furniture.

## Abbreviations

CONSORT: Consolidated Standards of Reporting Trials
FGD: focus group discussion
HEMV: health education and movement video
IDI: individual depth interview
IMU: inertial measurement unit
NCD: noncommunicable disease
RCT: randomized controlled trial
SB: sedentary behavior

## Data Availability

The datasets used and analyzed during the study are available from the corresponding author upon reasonable request.
