# Supplementary figures and images for: A Classroom-Based Intervention for Reducing Sedentary Behavior and Improving Spinal Health: Pragmatic Stepped-Wedge Feasibility Randomized Controlled Trial
Source: JMIR Form Res. 2025 Feb 24;9:e65169. doi: 10.2196/65169 (PMC11894349; doi:10.2196/65169)

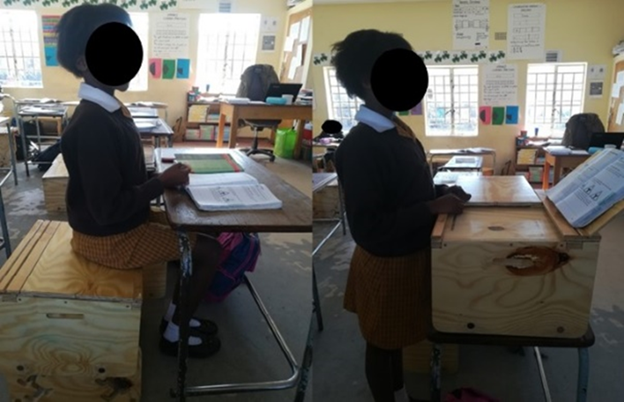

Supplement: Multimedia Appendix 2 [file formative_v9i1e65169_app2.png]

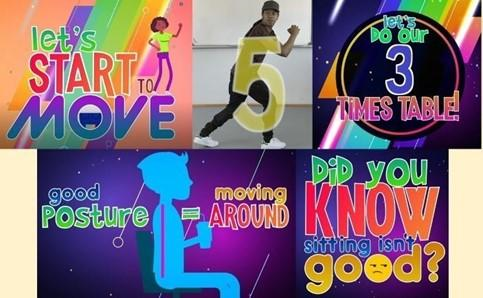

Supplement: Multimedia Appendix 3 [file formative_v9i1e65169_app3.png]

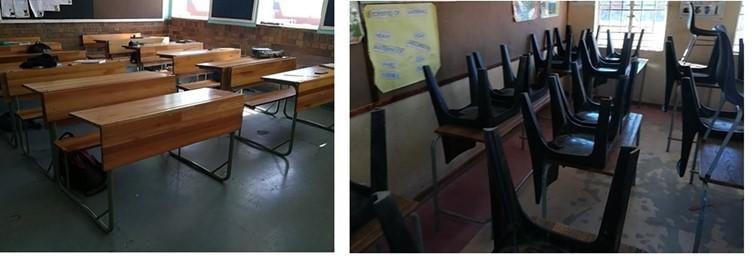

Supplement: Multimedia Appendix 4 [file formative_v9i1e65169_app4.png]
